# Characterization and Biological Activity of Taishan *Pinus massoniana* Pollen Polysaccharide *In Vitro*

**DOI:** 10.1371/journal.pone.0115638

**Published:** 2015-03-17

**Authors:** Shifa Yang, Kai Wei, Fengjuan Jia, Xue Zhao, Guolin Cui, Fanxia Guo, Ruiliang Zhu

**Affiliations:** Shandong Provincial Key Laboratory of Animal Biotechnology and Disease Control and Prevention, College of Animal Science and Technology, Shandong Agricultural University, Shandong, Taian, 271018, PR China; St. Jude Children's Research Hospital, UNITED STATES

## Abstract

Taishan *Pinus massoniana* pollen polysaccharide (TPPPS) improves cellular and humoral immune responses of animals and is a novel potential immunomodulator. However, the components of TPPPS have not been recognized. To investigate the composition of TPPPS, crude polysaccharide was obtained from Taishan *P*. *massoniana* pollen through water extraction and ethanol precipitation. Three homogeneous polysaccharide fractions (TPPPS1, TPPPS2, and TPPPS3) were purified from TPPPS by DEAE-cellulose column chromatography. The average molecular weights of the three polysaccharides were 56, 25, and 128 kDa, respectively. Results of high-performance liquid chromatography (HPLC) showed that TPPPS comprised mannose, ribose, xylose, glucuronic acid, galacturonic acid, glucose, galactose, and arabinose. The biological activity assays showed that TPPPS2 and TPPPS3 significantly promoted spleen lymphocyte proliferation, and that TPPPS3 showed better effect than TPPPS2. TPPPS3 enhanced the secretion of cytokine IL-2 and TNF, whereas TPPPS2 mainly elevated IL-2 secretion. By contrast, TPPPS1 exhibited other effects, and it induced the highest amount of NO production, thereby indicating that TPPPS1 had the best antioxidant activity. TPPPS3 at 50 μg/mL significantly inhibited the proliferation of subgroup B Avian Leukosis virus (ALV-B) through virus adsorption interference *in vitro*. Results indicated that TPPPS comprised three main components, among which, TPPPS1 mainly showed antioxidant effects, whereas TPPPS2 and TPPPS3 played key roles in immunomodulation, especially TPPPS3. Further studies on the use of a reasonable proportion of TPPPS1-3 may facilitate the development of an effective immunomodulator.

## Introduction

Polysaccharide, an important biomacromolecule of organisms, has a highly complex structure and species specificity. It is a critical factor in cell surface signal identification, antigen-antibody reaction, and intercellular signal transmission and perception [1, 2]. A growing number of studies have corroborated that polysaccharides from natural plants could significantly improve the function of the immune system and promote the self-protection of the body [3–5]. Luo et al. reported that polysaccharides from the stems of *Dendrobium officinale* significantly enhanced natural killer cell-mediated cytotoxicity and increased the phagocytosis and nitric oxide (NO) production of macrophages [6]. Another polysaccharide from *Astragalus* radix could increase the level of cytokines (TNF and GM-CSF) and the production of NO [7]. In addition, polysaccharides from fruit calyx of *Physalis alkekengi* var. *franchetii* showed increased scavenging effects on 1,1-dipheny-l-2-picrylhydrazyl and superoxide anion-scavenging activities [8]. Because of their effective biological activities, plant polysaccharides have been regarded as novel promising immunomodulatory agents, which are relatively nontoxic and with hardly any significant side effects [9–11].

Pollen has been used as dietary supplement in traditional medicine for centuries, and it has various functions, including alleviating fatigue, delaying apolexis, and treating disease. Pine pollen, known as the “King of Pollen” in China, is an omnipotent nutritional pollen [12]. Oral use of pine pollen dates back to the Tang Dynasty, which marked the beginning of the pine pollen industry [13]. Our previous studies have demonstrated that natural nontoxic polysaccharides derived from Taishan *Pinus massoniana* pollen (TPPPS) could enhance immunological function in mice, rabbits, and chickens [13–15]. Moreover, crude TPPPS, when used as an immunoadjuvant, could significantly improve the effects of different vaccines, such as *Proteus mirabilis*, the rabbit hemorrhagic disease, and the recombinant *Bordetella avium* ompA subunit vaccines [13, 15, 16]. However, until now the composition of TPPPS has not been determined, and the biological activity of each composition is also unknown.

In this study, we extracted the crude polysaccharides from Taishan *P*. *massoniana* pollen. Subsequently, three components of TPPPS (TPPPS1, TPPPS2, and TPPPS3) with molecular weights of 56, 25, and 128 kDa, respectively, were identified. The effects of TPPPS1-3 on spleen lymphocyte proliferation, cytokine secretion, NO production, and virus propagation were determined as indices for evaluating *in vitro* biological activity. Our findings demonstrated that TPPPS2 and TPPPS3 had immunomodulatory properties, whereas TPPPS1 had good antioxidant activity. Notably, TPPPS3 could also inhibit viral infection by influencing virus adsorption.

## Methods and Materials

### Ethics Statement

Fresh Taishan *P*. *massoniana* pollen was collected from wild *P*. *massoniana* growing in a Taishan state-owned public area. No specific permissions were needed to collect Taishan *P*. *massoniana* pollen. Endangered or protected species were not included in our field studies. All animal procedures performed in this study were reviewed, approved, and supervised by Shandong Institute of Animal Husbandry and Veterinary (Permit No.: 2011749).

### Preparation and isolation of TPPPS

Taishan *P*. *massoniana* pollen, collected from the Taishan region in China, was sieved through a 260 mesh, and pulverized by Ultra-Micro Pulverizer from Taian Zhengxin Science and Technology Co., Ltd., Shandong, China. TPPPS was extracted by water extraction and ethanol precipitation according to the procedure described by Wei et al. [13]. The yield and purity rates of TPPPS were calculated and detected using phenol-sulfuric acid method [17]. The crude polysaccharide was dissolved in distilled water and applied to a DEAE-cellulose column (700 mm × 15 mm) (Xiamei, China). The column was initially eluted with distilled water, 0.01, 0.03, 0.05, 0.1, 0.3, and 0.5 M NaCl, and 0.5 M NaOH at a flow rate of 0.8 mL/min. Three peaks of polysaccharide fractions were collected with a fraction collector and concentrated using a rotary evaporator at 55°C. The three peaks were designated as TPPPS1, TPPPS2, and TPPPS3 respectively. TPPPS1, TPPPS2, and TPPPS3 (collectively designated as TPPPS1-3) were further purified with a Sephadex G-200 gel column (700 mm × 15 mm) and eluted with 0.1 M NaCl at a flow rate of 0.2 mL/min. The major fraction was collected and subsequently freeze dried.

A Super-Bradford Protein Assay Kit (CWBIO, China), which was based on the Coomassie brilliant blue G-250 method, was used to determine the protein concentrations in TPPPS and TPPPS1-3.

### Molecular weight (Mw) and monosaccharide composition of TPPPS1-3

The homogeneity and Mw of TPPPS1, TPPPS2, and TPPPS3 were measured by high-performance gel-permeation chromatography (HPGPC) [18]. The sample solution (20 μL of 0.5%) was applied to the column (7.8 mm × 300 mm, 5 μm), eluted with 0.05 M Na_2_SO_4_ solution at a flow rate of 0.5 mL/min, and detected by an RID-10A refractive index detector. Light scattering data from MALLS were processed with the ASTRA software.

HPLC analyses were performed to determine the monosaccharide composition [19]. TPPPS1, TPPPS2, and TPPPS3 (1 mg) were hydrolyzed with trifluoroacetic acid and derivatized with 1-phenyl-3-methyl-5-pyrazolone (PMP) and NaOH. Subsequently, HCl was added for neutralization, and extraction was performed thrice with chloroform. The standard monosaccharides included mannose (Man), ribose (Rib), xylose (Xyl), glucuronic acid (GluA), galacturonic acid (GalA), glucose (Glc), galactose (Gal), and arabinose (Ara) (Dr. Ehrenstorfer GmbH, Germany). These standard monosaccharides were also derivatized with PMP using the same method as that used for the derivatization of TPPPS1-3. HPLC conditions were as follows: Column, C18, 150 mm × 4.6 mm, 5 μm (Shimadzu, Japan); column temperature, 40°C; mobile phase, 0.05 M KH_2_PO_4_ (pH 6.8) with 15% (solvent A) and 40% (solvent B) acetonitrile in water; injection volume of 10 μL; and running time, 60 min. The elution gradient of solvent B was 0, 5, 10, and 60 min, and the corresponding concentration gradient was 10%, 11.6%, 14%, and 20%, respectively.

### Animals and cells

Specific-pathogen-free (SPF) chickens, 8 weeks old, were purchased from Sipafasi Poultry Co., Ltd. Jinan, China. Chickens were kept in filtered air positive pressure isolation units (Lingshu, China). Splenocytes were cultured in RPMI-1640 medium (GBICO, USA) supplemented with 10% fetal bovine serum (GBICO, USA) and 100 IU/mL penicillin in a 95% humidified atmosphere containing 5% CO_2_ at 37°C.

### Splenocyte proliferation and cytokine detection

Spleen from the SPF chicken was removed to prepare splenocytes, as described by Chen et al. [20]. Cell suspensions were prepared after the lysis of erythrocytes in RBC lysing buffer (Sigma, USA) and adjusted to 2 × 10^6^ cells/mL to 10 × 10^6^ cells/mL in RPMI-1640 medium containing 10% fetal calf serum. Splenocytes were distributed (100 μL per well) on 96-well plates (Costar Products, USA) and cultured with different concentrations of TPPPS and TPPPS1-3 (6.25, 12.5, 25, 50, and 100 μg/mL) for 72 h. Splenocyte proliferation activity was tested by 3-(4, 5-dimethylthiazol-2-yl)-2, 5-diphenyl tetrazolium bromide (MTT; Sigma, USA) assay according to the method of Iribe [21]. The stimulus index (SI) was calculated by the following equation: SI = OD_experimental_/OD_control_

Splenocytes were cultured with different concentrations of TPPPS and TPPPS1-3 for 24 h using the proliferation assay method. The supernatants were collected to detect IL-2 and TNF production using commercial ELISA kits according to the manufacturer’s instructions (Mlbio, China).

### NO production

NO, quantified indirectly by nitrite accumulation in the culture medium, was measured spectrophotometrically using the Griess reaction, with NaNO_2_ as the standard [22]. Splenocytes (1 × 10^6^ cells/well) were incubated in 96-well plates, and different concentrations (6.25, 12.5, 25, 50, and 100 μg/mL) of TPPPS and TPPPS1-3 were added to wells at 37°C for 24 h. The wells containing the same amount of PBS served as the control group. Subsequently, 100 μL aliquots of the supernatant were mixed with 100 μL of 1% sulfanilamide in 50% H_3_PO_4_. After 5 min at room temperature, 100 μL of 0.1% N-1-naphthylenediamine was added. The nitrite concentration was determined 15 min later at 570 nm [23].

### Cells and viruses

DF1 cells, which comprise a continuous cell line of chicken embryo fibroblasts [24], were grown in DMEM supplemented with 10% fetal calf serum. For the maintenance medium (MM), the serum concentration was reduced to 1%.

ALV-B virus strain was used to determine the antivirus activity of TPPPS and TPPPS1-3. Virus titers were calculated by the TCID_50_ method using Avian Leukosis Virus Antigen Test ELISA kits (IDEXX, USA).

### Cytotoxicity assay

Cell viability was measured by MTT assay. Cell suspensions were seeded into 24-well plates (1 × 10^5^ cells/well) for 4 h. Subsequently, the medium was supplied with different concentrations (1, 10, 100, 1000, and 5000 μg/mL) of TPPPS and TPPPS1-3. After 24 h incubation at 37°C and 5% CO_2_, 10 μL of MM containing MTT (at a final concentration of 0.5 mg/mL) was added to each well for 4 h. After 4 h of incubation at 37°C, cell viability was tested by MTT test [25].

### Antiviral assay

Antiviral activity was evaluated by TCID_50_ assay on DF1 cells. Briefly, DF1 cell monolayers grown in 24-well plates were infected with 10 μL (TCID_50_ of 10^3.75^/0.1 mL) ALV-B in the absence or presence of various concentrations (6.25, 12.5, 25, 50, and 100 μg/mL) of TPPPS or TPPPS1-3. After 1 h adsorption at 37°C, residual inoculum was replaced by MM containing the corresponding dose of TPPPS or TPPPS1-3. TCID_50_ was determined by ELISA kit after 9 d of incubation (IDEXX, USA). All experiments were performed in triplicate.

To investigate the reaction time of polysaccharides in the antiviral process, DF1 cells grown in 24-well plates were infected with ALV-B at 10 μL/well (TCID_50_ of 10^3.75^/0.1 mL) via three different polysaccharide treatment methods, as follows:

Adsorption (Ad): Cells were exposed to approximately 100 μL of the virus mixture, MM, and polysaccharides for 1 h at 4°C. After removal of the solution mixture, the cells were washed twice with PBS and covered with MM.

After adsorption (AA): Cells were infected with ALV-B in the absence of the polysaccharides. After adsorption for 1 h at 4°C, the non-adsorbed virus was removed. The cells were washed twice with PBS and subsequently incubated with MM containing polysaccharides.

Always (Al): Cells were infected with ALV-B in the presence of polysaccharides. After 1 h at 4°C, both polysaccharide and non-adsorbed virus were removed, and the cells were washed with PBS and overlaid with MM containing polysaccharides.

### Data analysis

All data were expressed as means ± standard deviations (SD) of three replicated values. SPSS 8.0 software (SPSS, USA) was used for data analysis. Data were analyzed by one-way ANOVA, followed by Duncan’s multiple-range tests to show the differences between groups. A *P*-value of <0.05 was considered statistically significant.

## Results

### Mws and monosaccharide compositions of TPPPS1-3

**Fig 1:**
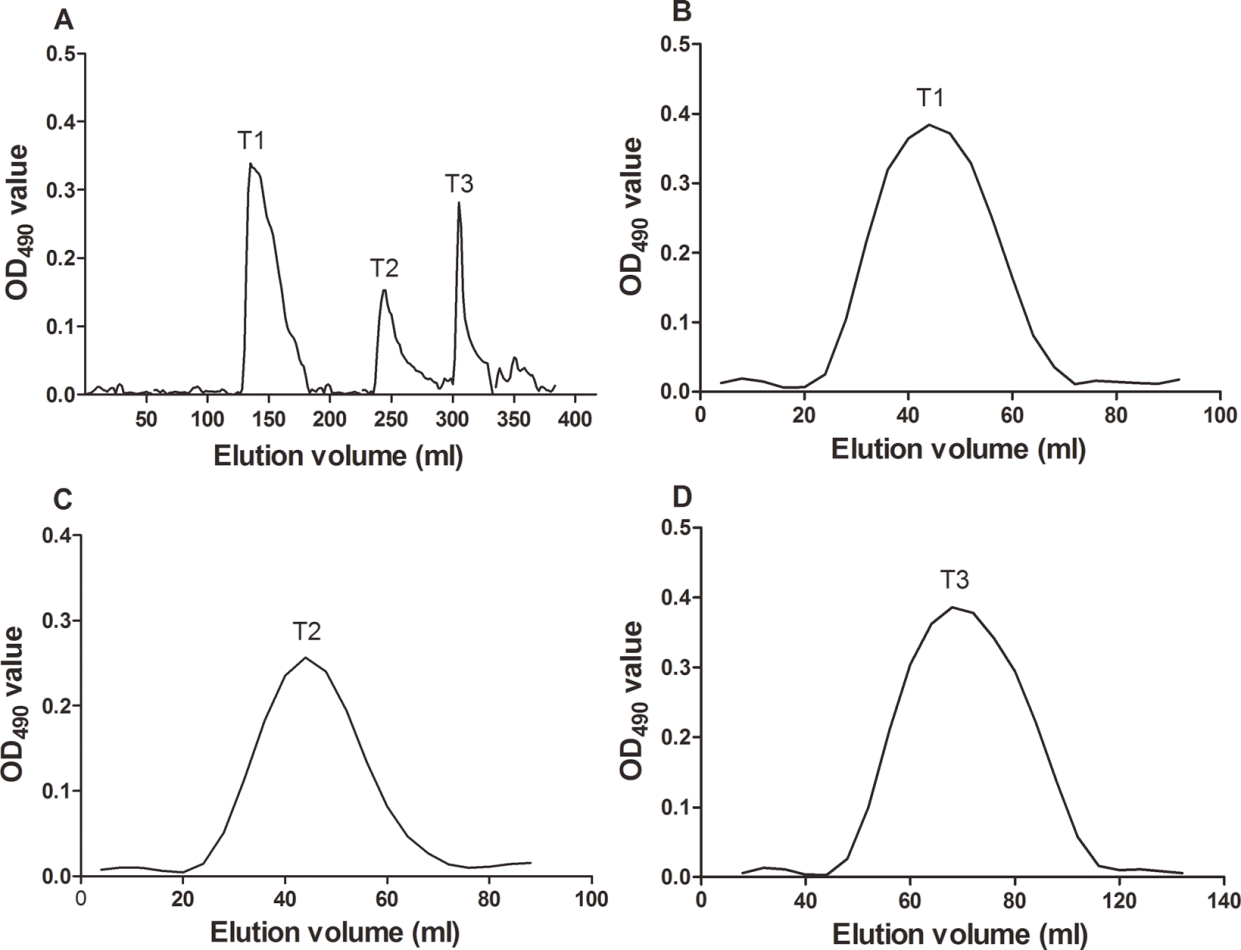
Isolation and purification of polysaccharides. (A) Ion exchange chromatogram of TPPPS on DEAE-cellulose column, eluted with distilled water, 0.01, 0.03, 0.05, 0.1, and 0.3 M NaCl. T1: TPPPS1; T2: TPPPS2; T3: TPPPS3. (B, C, and D) Gel filtration chromatogram of TPPPS1, TPPPS2, and TPPPS3, respectively, on Sephadex G-200 column eluted with 0.1 M NaCl.

Optimized hot water extraction and ethanol precipitation methods were performed to extract the polysaccharide from Taishan *P*. *massoniana* pollen in this study. In the process of extraction, proteins and lipids were eliminated to the maximum degree to obtain TPPPS with high purity. The yield rate of TPPPS was 3.12%, and the purity rate was 87.4%. To study the fractions of TPPPS, the ion-exchange chromatography of TPPPS on a DEAE-cellulose column (700 mm × 15 mm) was performed. As shown in Fig. 1, chromatography of TPPPS resulted in three peaks from distilled water and NaCl elution, and the peaks were designated as TPPPS1 (eluted with distilled water), TPPPS2 (eluted with 0.03 M NaCl), and TPPPS3 (eluted with 0.1 M NaCl). These three fractions were further purified by size-exclusion chromatography on a Sephadex G-200 column, and the three homogeneous fractions were obtained.

**Fig 2:**
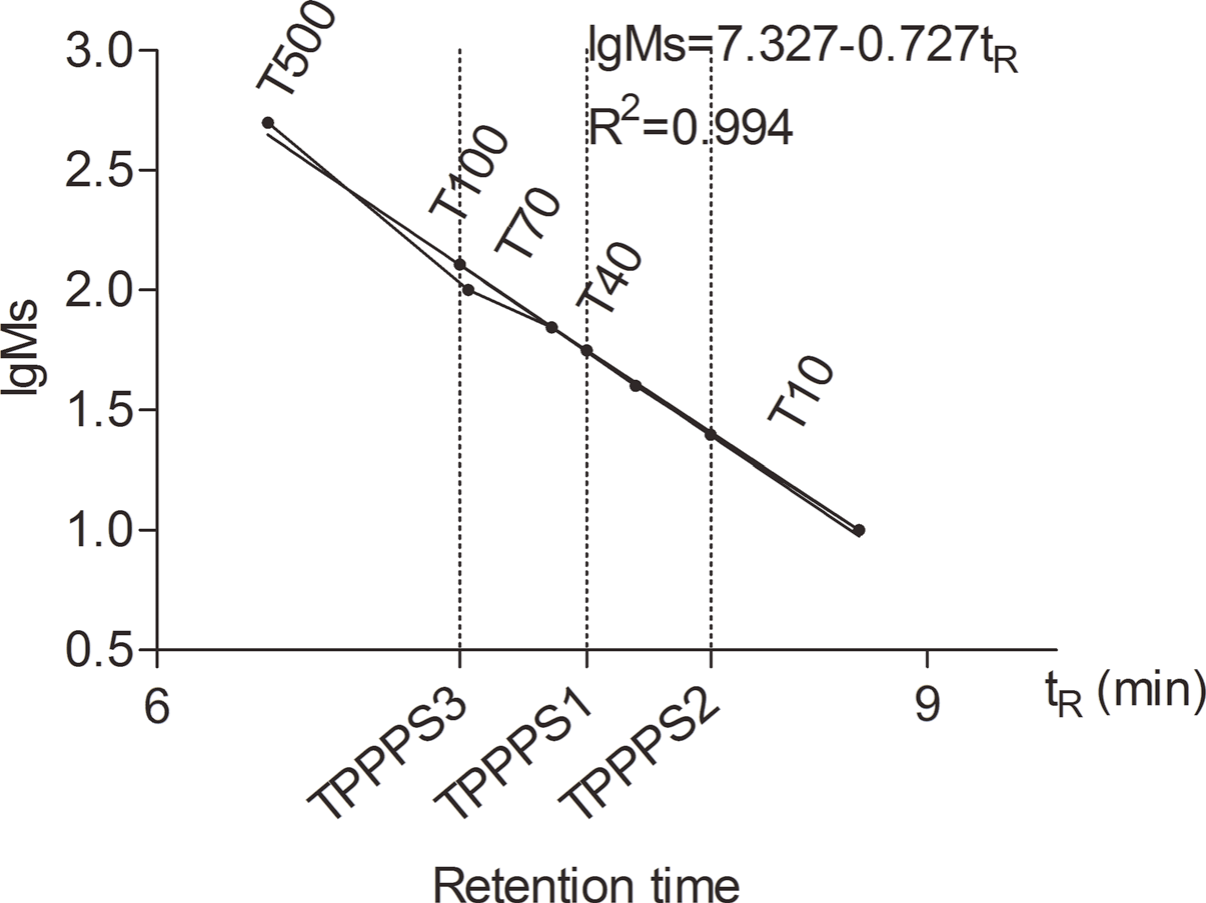
Mw determination with HPGPC system for TPPPS1-3. The standard curve was calibrated with T-series dextran (T-500, T-100, T-70, T-40, and T-10) as standard. lgMs = 7.327–0.727 t_R_, R^2^ = 0.994. lgMs was defined as the logarithm of the Mw, and t_R_ was the retention time. The molecular weight of TPPPS1-3 was estimated by the standard calibration curve.

According to the retention time and the calibration curve (lgMs = 7.327–0.727Tr, r^2^ = 0.994) made from a Dextran T series standard of known Mws (T-500, T-200, T-70, T-40, T-20, and T-10), the Mws of TPPPS1, TPPPS2, and TPPPS3 were estimated to be 56, 25, and 128 kDa, respectively (Fig. 2). HPLC analysis showed that the three fractions comprised mannose, ribose, xylose, glucuronic acid, galacturonic acid, glucose, galactose, and arabinose (Table 1) The most abundant monosaccharides in TPPPS1, TPPPS2, and TPPPS3 were ribose (21.5%), xylose (26.2%), and arabinose (26.3%), respectively. However, no xylose, ribose, and galactose were observed in TPPPS1, TPPPS2, and TPPPS3, respectively (Table 1).

**Table 1:** Components of monosaccharide from TPPPS1-3.

	Monosaccharide components^a^ (mol%)							
	Man	Rib	Xyl	GluA	GalA	Glc	Gal	Ara
TPPPS1	13.5%	21.5%	nd^b^	14.2%	3.8%	20.1%	14.6%	12.3%
TPPPS2	16.5%	nd	26.2%	9.8%	7.9%	19.9%	5.3%	14.4%
TPPPS3	2.9%	13.5%	18.5%,	15.2%	10.3%	13.3%	nd	26.3%

^a^ Man, mannose; Rib, ribose; Xyl, xylose, GluA, glucuronic acid; GalA, galactose acid; Glc, glucose; Gal, galactose; Ara, arabinose.

^b^ nd, not detected.

### Effect of TPPPS 1-3 on splenocyte proliferation and cytokine secretion

Prior to the experiment, the protein concentrations in Taishan *P*. *massoniana* pollen, TPPPS, and TPPPS1-3 were determined using Super-Bradford Protein Assay Kit (CWBIO, China) to evaluate the purity of polysaccharides. The results showed that the protein content in Taishan *P*. *massoniana* pollen was 16.89%, whereas that in TPPPS was merely 0.32% after treatment with pepsin and Sevage reagent. Furthermore, after purification with a Sephadex G-200 gel column, the protein contents of TPPPS1-3 were 0.01%, 0.02%, and 0.02%, respectively.

**Fig 3:**
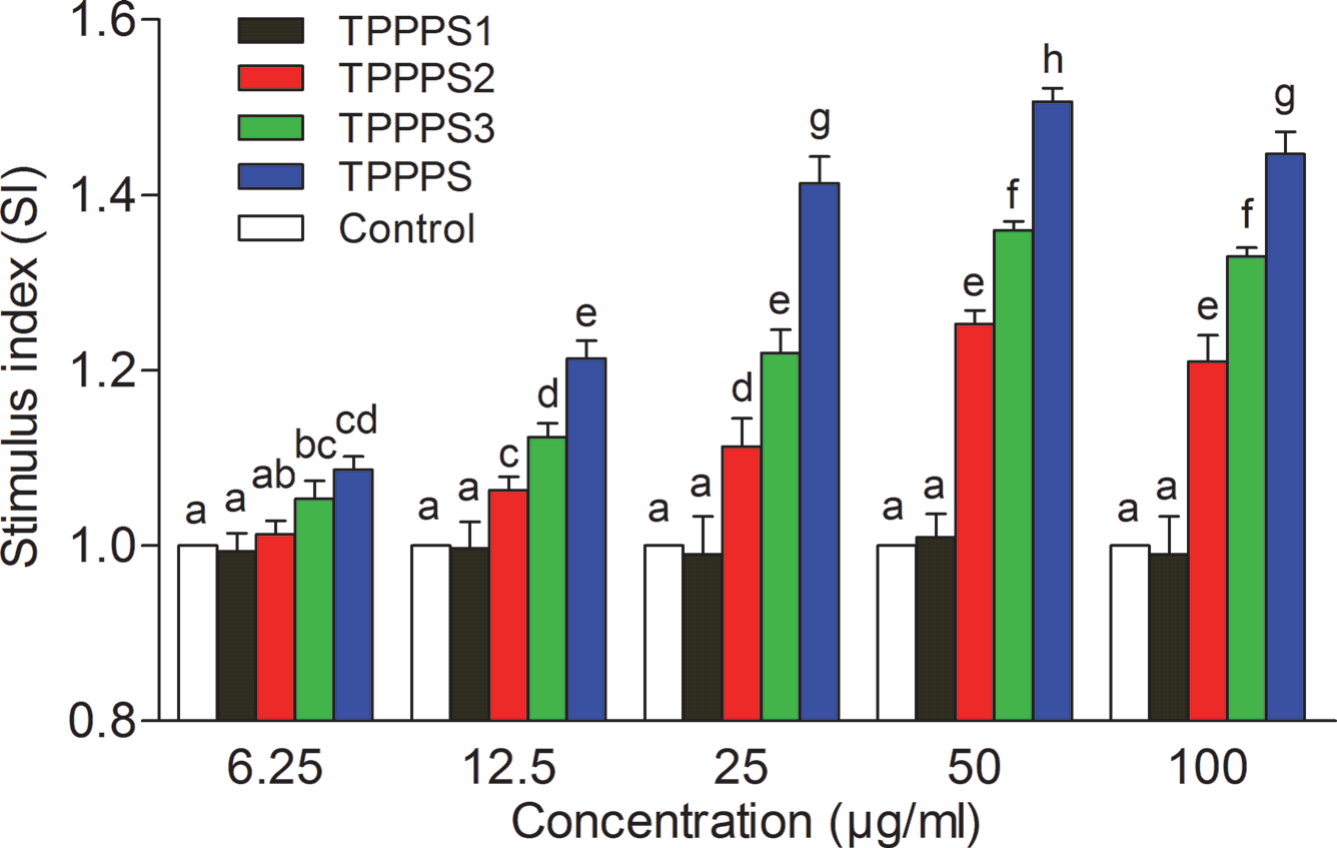
Effects of TPPPS1-3 and TPPPS on splenocyte proliferation *in vitro*. Splenocytes were isolated and cultured in 96-well plates. After ConA stimulation, the proliferation was examined by MTT method as described in the Materials and Methods. Stimulus index represents the ratio of absorbance between the experimental group and control group, and the values are presented as mean ± SD from five independent experiments. Different superscripts indicate a significant difference (*P* < 0.05).

Spleen is an important nonspecific peripheral lymphoid organ, which is the major site of immune response to blood borne antigens [26]. To investigate the immunomodulatory activity of TPPPS1-3, we first determined their effects on splenocyte proliferation. The crude TPPPS served as the control. As shown in Fig. 3, TPPPS3 and the crude TPPPS promoted splenocyte proliferation at 6.25 μg/mL, and TPPPS2 significantly promoted the proliferation of splenocytes at 12.5 μg/mL (*P* < 0.05). However, TPPPS1 failed to exhibit any effect on splenocyte proliferation compared with the control. This result indicated that TPPPS3 was the most effective among the fractions for enhancing splenocyte proliferation. The activity of TPPPS3 was better than that of TPPPS2. Crude TPPPS had the best stimulatory effect on the proliferation of splenocytes at all concentrations, and the highest effect was obtained at 50 μg/mL.

**Fig 4:**
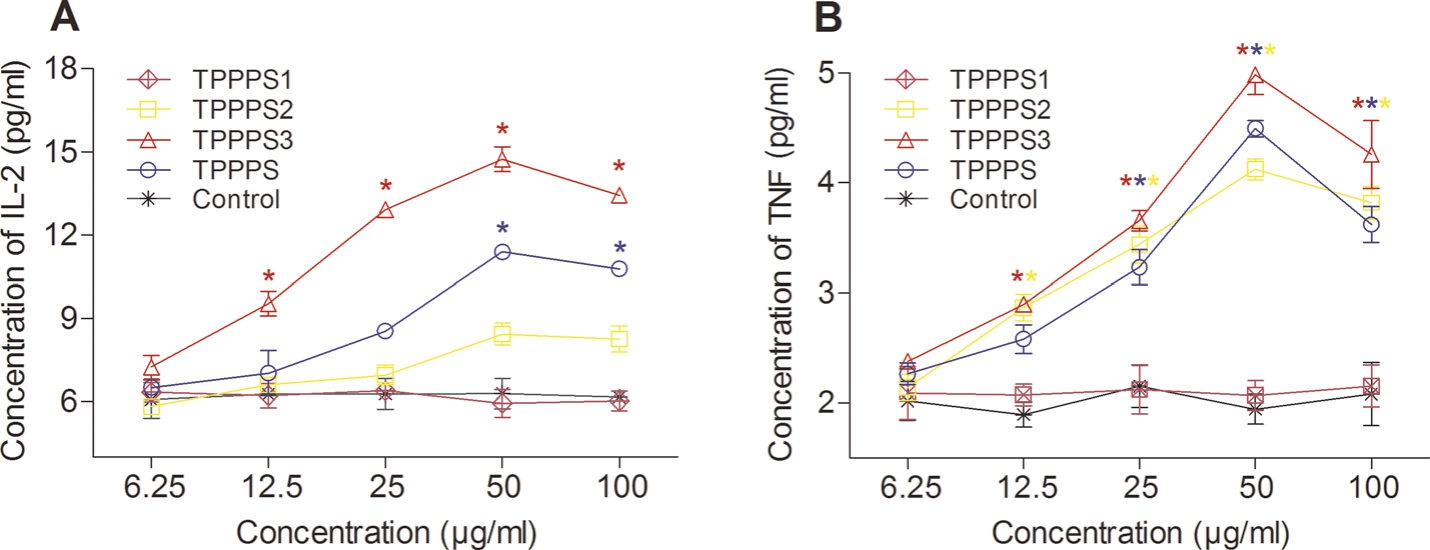
Effects of TPPPS1-3 and TPPPS on IL-2 and TNF secretion *in vitro*. Activated splenocytes in 96-well plates were treated with different concentrations of TPPPS1-3 and TPPPP, then IL-2 and TNF in the supernatant was detected by ELISA. PBS treatment wells served as the control, and the values are presented as mean ± SD of five independent experiments. An asterisk indicates that the value of the corresponding group was significantly different from that of the control group (*P* < 0.05, ANOVA).

Cytokines are crucial for fighting off infections and are involved in immune responses [27]. The effect of TPPPS1-3 on the production of cytokine secretions (IL-2 and TNF) from spleen cells *in vitro* is shown in Fig. 4. Increased IL-2 and TNF secretions were observed in TPPPS2, TPPPS3, and the crude TPPPS groups. Like the control group, TPPPS1 showed no effect on the secretion of these two cytokines. Among the three polysaccharide fractions, TPPPS3 significantly increased the IL-2 and TNF secretions (*P* < 0.05), and the optimal concentration was 50 μg/mL. TPPPS2 significantly increased TNF secretion (*P* < 0.05) and slightly increased IL-2 secretion.

### Effects of TPPPS1-3 on NO production

**Fig 5:**
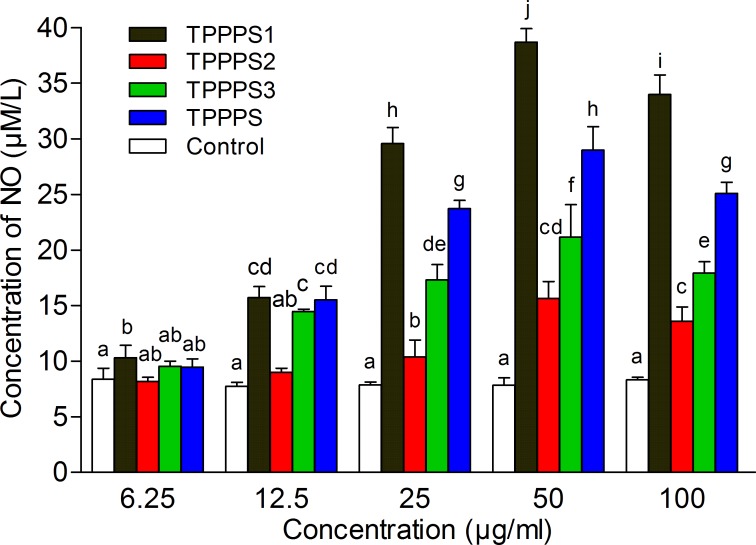
Effects of TPPPS1-3 and TPPPS on production of NO *in vitro*. The supernatant in 96-well plates cultured with splenocytes were collected. NO production was measured spectrophotometrically using the Griess reaction. PBS treatment wells served as the control, and the values are presented as mean ± SD from five independent experiments. Different superscripts indicate a significant difference (*P* < 0.05).

To further test the antioxidant activity of TPPPS1-3, the NO production of splenocytes treated with polysaccharides was measured. We observed that TPPPS and its three fractions increased the NO production of splenocytes, and the best effects were obtained at 50 μg/mL (Fig. 5). However, the effects of different fractions on the stimulation of NO production varied, and TPPPS1 group obviously exhibited the highest value, followed by TPPPS, TPPPS3, and TPPPS2. Notably, NO production under TPPPS1 treatment was significantly higher than under the other treatment groups at 25, 50, and 100 μg/mL (*P* < 0.05). Moreover, TPPPS, TPPPS1, and TPPPS3 significantly elevated NO production at a low concentration (12.5 μg/mL), whereas TPPPS2 induced NO production at a concentration greater than 25 μg/mL. These results suggested that TPPPS1 showed the most effective antioxidant activity, but it failed to enhance splenocyte proliferation.

### Antiviral activity of TPPPS1-3

Initially, a cytotoxicity assay was performed to determine the side effects of these four polysaccharides on chicken embryo fibroblasts. The result showed that TPPPS and TPPPS1-3 at concentrations ranging from 1 μg/mL to 5 mg/mL had no significant toxicity and side effects on the activity of chicken embryo fibroblasts.

**Fig 6:**
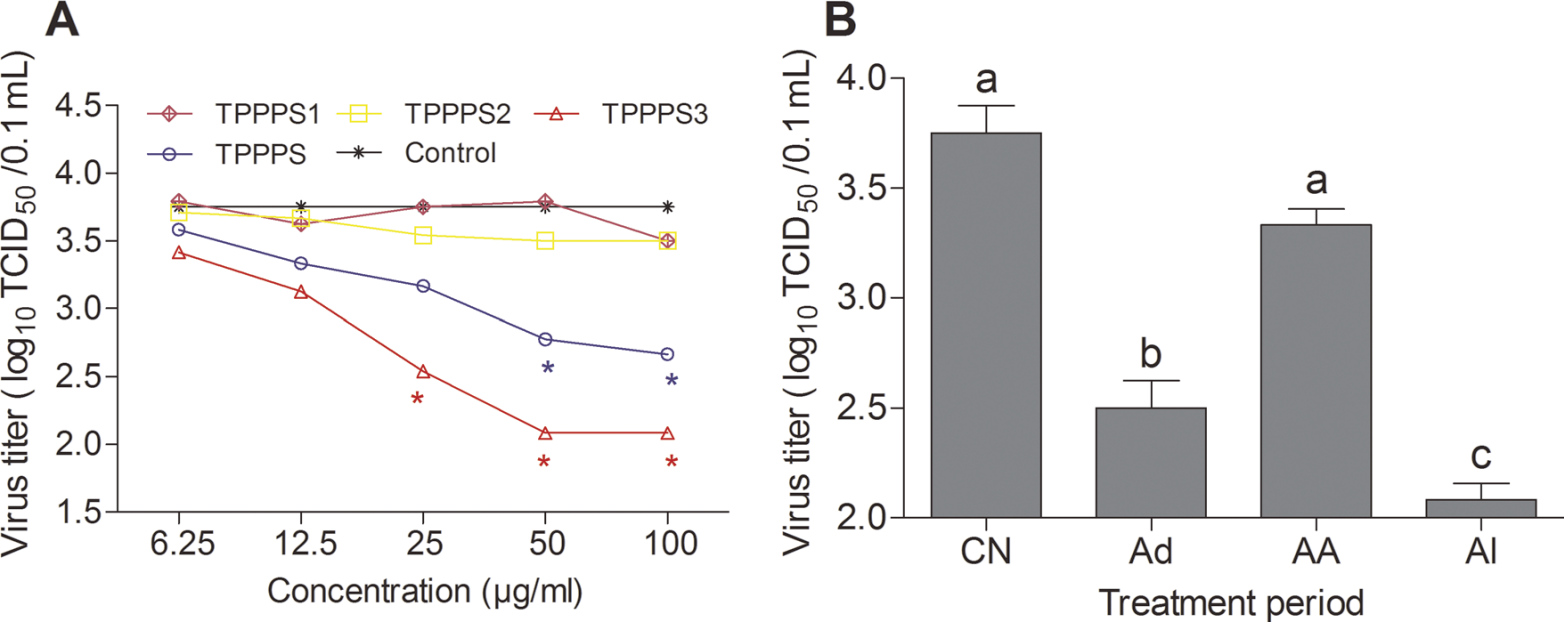
Effects of TPPPS1-3 and TPPPS on antiviral activity *in vitro*. (A) DF1 cells cultured in 24-well plates were infected with 10 μL/well (TCID_50_ of 10^3.75^/0.1 mL) ALV-B. After 9 d of culture in MM containing different concentrations of TPPPS or TPPPS1-3, viral titers in the supernatant were directly determined by TCID_50_ assays on DF1 cells. PBS treatment wells served as the control. All values shown are presented as means ± SD from three independent experiments. An asterisk indicates that the value of the corresponding group was significantly different from that of the control group (*P* < 0.05, ANOVA). (B) DF1 cells infected with ALV-B were treated with 50 μg/mL of TPPPS3 at different viral infection periods, and viral titers in the supernatant were directly determined by TCID_50_ assays on DF1 cells. Ad is defined as the “adsorption” period; AA is defined as the period “after adsorption”; Al is the period wherein the polysaccharide was always present. PBS treatment wells served as the control. All values shown are presented as means ± SD from three independent experiments. Different superscripts indicate a significant difference (*P* < 0.05).

An antiviral activity assay was conducted according to the method described in Methods and Materials. TPPPS3 at 50 μg/mL showed the best inhibitory effect on ALV-B on chicken embryo fibroblasts among the fractions (*P* < 0.05) (Fig. 6A). Therefore, TPPPS3 at 50 μg/mL was selected for the detection of the polysaccharide reaction time. As illustrated in Fig. 6B, in the viral “Ad” period, the TCID_50_ of ALV-B was significantly lower than in the “AA” and in the control treatment. Additionally, the TCID_50_ of ALV-B in the viral “Ad” period was slightly higher than in the “Al” period, thereby suggesting that TPPPS3 antiviral activity mainly occurred during viral adsorption. Moreover, the TCID_50_ of ALV-B was the lowest during the “Al” period, thereby indicating that TPPPS3 could obviously inhibit virus proliferation during the whole incubation period.

## Discussion

Many polysaccharides isolated from plant and microorganism have been studied in the biomedical field, and these polysaccharides represent an unlimited resource because of their various biological activities [28]. Our recent studies have shown that TPPPS is an excellent immunomodulator, and it can enhance and improve the immune status of animals. However, the active ingredients of TPPPS are largely unknown. In the current study, we preliminarily investigated the active components of TPPPS and their respective bioactivities. We found three polysaccharides (TPPPS1-3), which are the major components of TPPPS. These three different components exhibited different biological functions, including immunomodulation, antioxidation, and antiviral activities.

The crude TPPPS was extracted from Taishan *P*. *massoniana* pollen. Three homogeneous fractions (TPPPS1, TPPPS2, and TPPPS3) were isolated and characterized. Polysaccharide is a macromolecular polymer, and its Mw is not only responsible for its physical properties (such as viscosity and solubility), but is also associated with biological activity. In this study, we determined the Mws of three fractions (TPPPS1-3) and analyzed their monosaccharide composition. Based on the results of the HPLC analysis, the Mws of TPPPS1, TPPPS2, and TPPPS3 were 56, 25, and 128 kDa, respectively. Generally, polysaccharides with higher Mws had a more complex structure and composition [29]. Here, various proportions and species of monosaccharide compositions in TPPPS1-3 were observed. We observed the absence of xylose, ribose, and galactose in TPPPS1, TPPPS2, and TPPPS3, respectively. Xylose is hard to be absorbed and utilized by some animals, including human, but this compound is involved in signal transduction between cells [30]. Ribose is a component of the RNA molecule, whereas galactose generally serves as nutrients of the cells. These obvious differences in the monosaccharide compositions of TPPPS1, TPPPS2, and TPPPS3 may contribute to the differences in their biological activities.

Lymphocytes play a key role in the adaptive immune response, and lymphocyte proliferation is relevant to the status of cellular and humoral immunity in organisms, in terms of protecting the organism against foreign agents and infectious diseases [31]. Spleen is the maximum peripheral lymphoid organs of birds and mammals, which contains large quantities of mature lymphocytes (B cells and T cells) and macrophages etc. In splenocyte proliferation assays, TPPPS exhibited the best effect on splenocyte activation among the fractions comprising TPPPS2 and TPPPS3 as effective components, and in particular, TPPPS3. These results indicated that TPPPS had strong immunomodulatory effects on splenocyte proliferation, and TPPPS2 and TPPPS3 may have synergistic effects.

Cytokines are important factors in regulating immune responses. TNF is mainly produced by macrophages which are important innate immunity cells, and it can improve non-specific immune function [32]. IL-2, which is mainly produced by helper T cells, can promote T cell differentiation and maturation, thereby enhancing the roles of B cells and macrophages. The secretions of TNF and IL-2 could indirectly reflect the levels of immune response. Numerous types of plant-derived polysaccharides can modulate the production of a range of cytokines in splenocytes [20, 33]. Our findings demonstrated that the production of T lymphocyte-related cytokine IL-2 could be significantly elevated by TPPPS3 treatment, whereas the secretion of macrophage-related cytokine TNF could significantly be increased by TPPPS2 and TPPPS3 treatments in a dose-dependent manner. These results suggested that both TPPPS2 and TPPPS3 could improve innate immunity, and TPPPS3 also enhanced adaptive immunity.

Additionally, nitrogen monoxide, a simple antioxidative molecule, is an important biological regulator and a fundamental component in the fields of neuroscience, physiology, and immunology [34–36]. In this study, TPPPS1 significantly produced a much higher amount of NO compared with TPPPS2 and TPPPS3. TPPPS1 could regulate NO production to contribute to the nonspecific host defense and inflammation, even if TPPPS1 failed to show immunomodulatory activity.

Various natural sources of polysaccharides show antiviral activity and low toxicity [37, 38]. Natural polysaccharides or polysaccharide derivatives with antiviral potentials have good application prospects as antiviral drugs. In this study, we found that TPPPS1–3 had no toxicity and side effects on the viability of DF1 cells, and that TPPPS3 exhibited potent inhibitory activity against ALV-B infection. Moreover, results of the virus yield reduction assay, which was performed under different polysaccharide treatment conditions (Ad, AA, Al, and control), revealed that the mechanism of TPPPS3 against ALV-B was through virus adsorption interference. In addition, we determined the reactivity of the fractions via *Limulus* amebocyte lysate (LAL) assay for endotoxin contamination and via hemagglutination assay for hemagglutination activity, and the results were negative (data not shown).

In summary, we identified three mainly fractions of TPPPS, and differences in the biological activity of these three TPPPS fractions were observed. Both TPPPS2 and TPPPS3 participated in the immune regulation process and were involved in improving splenocyte proliferation and cytokine secretion, but TPPPS3 showed better effects than TPPPS2. TPPPS3 also exhibited potent inhibitory activity against ALV-B infection. TPPPS1 exhibited the strongest antioxidant activity among the fractions, but we did not observe immunoregulatory activity for TPPPS1. In addition, we found that the effect of crude TPPPS was better than its fractions in enhancing splenocyte proliferation, indicating that TPPPS1-3 showed synergistic immunoregulatory effects. Our results contribute to the current knowledge on TPPPS, which can serve as a potential immunomodulator. A reasonable proportion of TPPPS1-3 may facilitate the development of a novel and effective immunomodulator.
